# Role of miRNA-Regulated Cancer Stem Cells in the Pathogenesis of Human Malignancies

**DOI:** 10.3390/cells8080840

**Published:** 2019-08-05

**Authors:** Abdul Q. Khan, Eiman I. Ahmed, Noor R. Elareer, Kulsoom Junejo, Martin Steinhoff, Shahab Uddin

**Affiliations:** 1 Translational Research Institute, Academic Health System, Hamad Medical Corporation, Doha, P.O. Box 3050, Qatar; 2 General Surgery Department, Hamad General Hospital, Hamad Medical Corporation, Doha, P.O. Box 3050, Qatar; 3 Department of Dermatology and Venereology, Hamad Medical Corporation, Doha, P.O. Box 3050, Qatar; 4 Weill Cornell Medicine, Doha, P.O. Box 24811, Qatar; 5 Weill Cornell University, New York, NY 10065, USA

**Keywords:** cancer, cancer stem cells, miRNA, signaling, human malignancies

## Abstract

Recent biomedical discoveries have revolutionized the concept and understanding of carcinogenesis, a complex and multistep phenomenon which involves accretion of genetic, epigenetic, biochemical, and histological changes, with special reference to MicroRNAs (miRNAs) and cancer stem cells (CSCs). miRNAs are small noncoding molecules known to regulate expression of more than 60% of the human genes, and their aberrant expression has been associated with the pathogenesis of human cancers and the regulation of stemness features of CSCs. CSCs are the small population of cells present in human malignancies well-known for cancer resistance, relapse, tumorigenesis, and poor clinical outcome which compels the development of novel and effective therapeutic protocols for better clinical outcome. Interestingly, the role of miRNAs in maintaining and regulating the functioning of CSCs through targeting various oncogenic signaling pathways, such as Notch, wingless (WNT)/β-Catenin, janus kinases/ signal transducer and activator of transcription (JAK/STAT), phosphatidylinositol 3-kinase/ protein kinase B (PI3/AKT), and **nuclear factor** kappa-light-chain-enhancer of activated B (NF-kB), is critical and poses a huge challenge to cancer treatment. Based on recent findings, here, we have documented the regulatory action or the underlying mechanisms of how miRNAs affect the signaling pathways attributed to stemness features of CSCs, such as self-renewal, differentiation, epithelial to mesenchymal transition (EMT), metastasis, resistance and recurrence etc., associated with the pathogenesis of various types of human malignancies including colorectal cancer, lung cancer, breast cancer, head and neck cancer, prostate cancer, liver cancer, etc. We also shed light on the fact that the targeted attenuation of deregulated functioning of miRNA related to stemness in human carcinogenesis could be a viable approach for cancer treatment.

## 1. Introduction

Cancer development or carcinogenesis is a complex and multistep phenomenon and involves accretion of an array of genetic, epigenetic, biochemical, and histological changes ultimately leading to the development of pathological manifestations. A number of etiopathological factors have been identified, and their role is well investigated. In spite of the significant advancement in the field of cancer research, obscurity to understand the real mechanism of cancer development is still there and thus requires a further in-depth investigation. Advancement of novel scientist friendly techniques has helped substantially in the understanding of cancer pathogenesis. Although cancer is originated from one mutated cell, it can grow to be highly heterogeneous, including various differentiated and proliferative cells with different markers. This heterogeneity is thought to be responsible for tumor development, relapse, metastasis, and therapeutic resistance. Among the several populations of cells accounting for the heterogeneity of cancer, the existence of cancer stem cells (CSCs) is highly important [1].

## 2. Normal and Cancer Stem Cells

Stem cells are undifferentiated cells, and they are classified into three types: adult stem cells, induced pluripotent stem cells (iPSCs), and embryonic stem cells (ESCs). Adult stem cells are named based on the tissue they are derived from, such as hematopoietic stem cells (HSCs), endothelial stem cells, mesenchymal stem cells (MSCs), cardiac stem cells, etc. ESCs are pluripotent stem cells produced from blastocyst’s inner cell mass or earlier morula phase embryos in epiblast tissue [2]. iPSCs are originated from somatic cell reprogramming and share several features of ESCs. Via differentiation, stem cells produce a mature cell. During embryonic development, ESCs can differentiate into any tissue type, while adult stem cells have significant roles in repairing and replenishing adult tissues [3]. Stem cells have huge potential in clinical treatment because of their self-renewal ability and pluripotency. Therapeutic importance of stem cells is also well elicited as findings revealed that stem cell transplantation has repaired injured organs [2].

A number of studies revealed that tumors contain a distinct subpopulation of cells which are highly tumorigenic and are involved in the progression of cancer. These cells are referred to as cancer stem cells (CSCs) and also known as tumor-initiating cells (TICs) [4,5]. The concept of cancer stem cells was initially established in the 1800s by Rudolf Virchow. In 1889, Steven Paget was the first to introduce the “seed and soil” hypothesis for cancer. He hypothesized that cells within a tumor could “seed” a metastasis in a remote organ which has favorable circumstances for the growth “soil” of a secondary tumor. Dick and colleagues isolated stem cells from leukemia for the first time in 1994, introduced the hypothesis that cancer tumors are formed hierarchically, and also confirmed its heterogeneity. CSCs are one of the main concerns related to cancer pathogenesis and drug development and are being recognized and isolated from several human malignancies [6]. Like normal cells, CSCs undergo cell division, growth, clonal expansion, and differentiation at an extensive rate to enlarge the stem cell population and development of characteristics to survive and evade the effect of cancer therapeutic measures [5]. Furthermore, CSCs have the ability of self-renewal, sphere formation, migration, invasion, and resistance to cancer therapy such as radiotherapy and chemotherapy, etc. Therefore, they are considered main contributors to the propagation of neoplastic cells. CSCs were initially discovered in leukemia; however, recent studies have demonstrated that CSCs play a role in the pathogenesis of several solid tumors, including prostate, brain, colon, pancreatic, and breast cancers. CSCs can originate from mutations in normal stem cells, from cancer cells which have endured epithelial-mesenchymal transition (EMT), or from dedifferentiated somatic cells. The molecular mechanisms underlying the development of CSC characteristics are still not well elucidated. However, recent findings revealed the role of microRNAs (miRNAs) in regulating CSCs [4]. Several cell surface markers have been identified and used to isolate CSCs, including CD24, CD133, CD44, and aldehyde dehydrogenase1 (ALDH1) [3]. It has also been evident that the markers, especially the surface markers of CSCs, are cancer-specific [7].

CSCs play a vital role in the development of tumor heterogeneity and acquired drug resistance. Although CSCs depict similarity with normal stem cells such as self-renewal and differentiation, interestingly, deregulation in the signaling pathways associated with the regulation of the basic stemness features accounts for the tumorigenic potential of CSCs [8,9]. Moreover, the development or the genesis of normal stem cells and associated functioning for tissue development is well elucidated while the origin of CSCs and its involvement in the maintenance and development of oncogenic potential for cancer pathogenesis is not well explored [10,11], and various hypothesis have been introduced about the genesis and functioning of CSCs.

**Figure 1:**
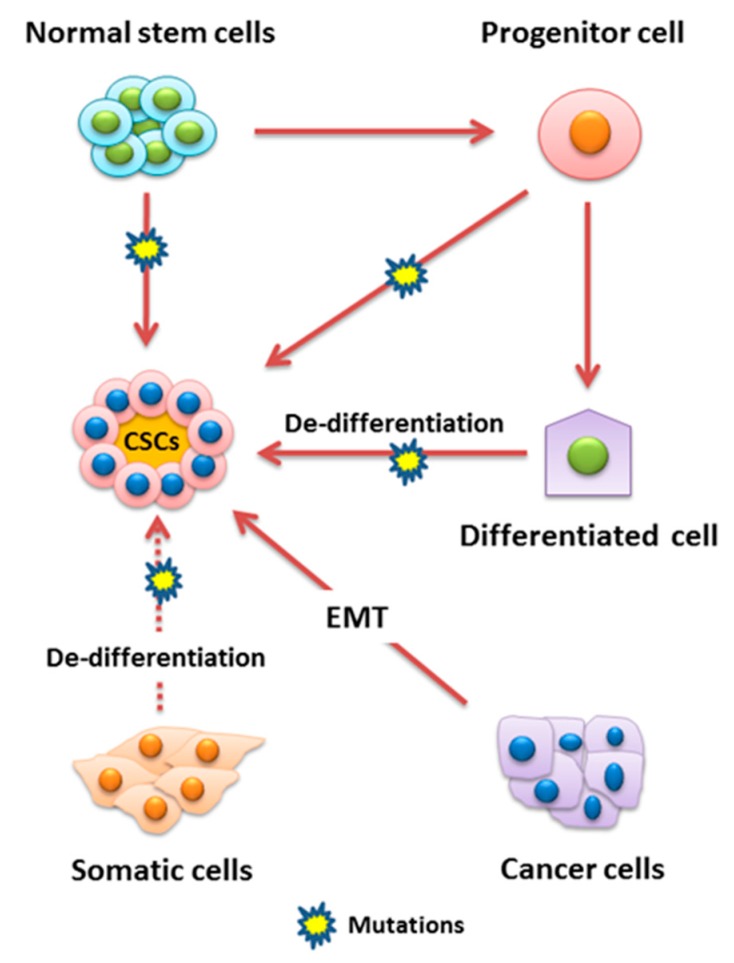
An overview of the origin of cancer stem cells (CSCs): CSCs can originate and develop from several cells. Normal stem cells may become CSCs through mutations, or it can develop into progenitor cells which in turn can develop into CSCs via mutations. In addition, progenitor cells can grow into CSCs through mutations and become a differentiated cell, which in turn can becomes undifferentiated and grow into CSCs. Moreover, CSCs can develop from dedifferentiated somatic cells or from cancer cells that undergo epithelial to mesenchymal transition (EMT).

The basic points of stem cell division theory suggest that cancer develops due to the accumulation of mutations in the genetic content of normal stem cells and that clonal expansion of these cells lead to the formation of CSCs and ultimately tumor formation and metastasis [12,13]. The principle concept of the CSC hypothesis explains the heterogenic nature of the tumor, i.e., tumor cells are not alike [14]. The CSC hypothesis key points include that only a minute number of cells inside a tumor have tumorigenic potential and can be separated from the rest of tumor cells. Furthermore, CSC-induced tumors contain a mixed population of tumorigenic and non-tumorigenic cells of the original tumor and can also be transplanted in various generations [15,16]. Thus, the overall concept is that CSCs have self-renewal and heterogenic differentiation potential, which results in tumor formation [14] (Figure 1). CSCs also arise due to a mutation in the differentiated normal cells and due to induced dedifferentiation, cells acquired stem-like properties from epigenetic reprogramming. Another variation between CSCs and normal stem cells involves stem cell niche compositions. Normal stem cell’s niches are tumor suppressive, consisting of several activated signaling pathways to arrest the growth. Conversely, CSC niches contain tumor microenvironmental cells including cancer-associated fibroblast that stimulate CSC growth and differentiation through growth factor secretion or activation of survival pathways through cell–cell interactions [4,9,13].

## 3. Cancer Stem Cells and Signaling Pathways

Most of the developmental biological phenomena and homeostasis are regulated by stem cells in a tightly controlled way by several regulatory mechanisms. A number of signaling pathways have been demonstrated to play a critical role in the regulation of distinct stem cell features including self-renewal, differentiation, cell fate decision, and survival. Particularly, the roles of Wnt (wingless)/β-catenin, Notch, tumor growth factor β (TGF-β), sonic hedgehog, Janus-activated kinase/signal transducer and activator of transcription (JAK/STAT), phosphatidylinositol 3-kinase/phosphatase and tensin homolog (PI3K/PTEN), and nuclear factor-kB (NF-kB), etc. have been well elucidated [17,18]. Decades of scientific findings demonstrate that most of the abovementioned signaling pathways are dysregulated in human malignancies, suggesting a critical role of CSCs in carcinogenesis of various malignancies such as colorectal, glioma, breast, pancreatic, leukemia, colon, and prostate cancers [17,19,20].

Deregulated Wnt/β-catenin signaling is one of the most extensively studied pathways critically associated with the development and survival of CSCs in various human malignancies [21,22,23]. Wnt/β-catenin is an evolutionary conserved signal transduction pathway vital for both initiation and regulation of a number of biological features such as stem cell self-renewal, calcium homeostasis, cell growth, polarity, migration, cell survival, and organogenesis [22,23]. Deregulated functioning of the Wnt/β-catenin pathway is associated with the pathogenesis of most human diseases including cancer, neurodegenerative, endocrine, inflammatory, and bone disorders [24]. Extensive work revealed the critical involvement of Wnt/β-catenin deregulated signaling in human malignancies including breast and colorectal cancers. The increased frequency of mutation in the signaling component of the Wnt/β-catenin pathway is detected both in solid and hematological malignancies [24]. Further, Wnt signaling is also critical for tumor cells to acquire CSC-like features through dedifferentiation. As deregulated Wnt signaling is implicated in most of the developmental and tumorigenic features of CSCs, targeting of Wnt would be of greater clinical relevance.

Another mechanism which is central for the growth and maintenance of CSCs is Hedgehog (Hh) signaling. The importance of Hh in various developmental and biological phenomena has been well elucidated. Additionally, the deregulated functioning of Hh has been widely reported in the cellular and molecular changes of carcinogenesis, including the survival of CSCs [25].

Notch signaling is another highly conserved signaling pathway well-known for the regulation of cell proliferation, differentiation, angiogenesis, stem cell fate determination, and development [26,27]. Accumulating findings reveal that deregulated Notch signaling is critically associated with the proliferation, differentiation, maintenance, angiogenesis, and migration of CSCs in human cancers [28,29]. Thus, targeting the Notch pathway and its regulator may be of great relevance in cancer management and eradication of CSCs. Another pathway implicated in CSCs is JAK/STAT signaling. A number of findings showed that JAK/STAT3 signaling pathway and its associated genes are abnormally activated in CSCs suggesting its important role in the maintenance of CSCs. In addition, STAT3 was reported to be the most upregulated gene in JAK/STAT3 signaling in breast CSCs [30]. PI3K signaling is tightly conserved, and it is associated with the regulation of several cellular processes such as growth, survival, and progression of the cell cycle. Moreover, PI3K signaling has been shown to be critical for growth and proliferation of normal and stem cells [17]. NF-κB (nuclear factor kappa-light-chain-enhancer of activated B cells) is an important transcription factor that plays a vital role in various biological and pathological conditions such as cell growth and proliferation, inflammation, apoptosis, angiogenesis, and cell migration [31,32]. Aberrant or uncontrolled activation of NF-KB has been well-linked with most of the human pathological conditions including cancer [33,34]. A number of findings demonstrated the critical role of NF-KB in the maintenance, expansion, proliferation, and survival of CSCs [35]. Thus, targeted attenuation of the deregulated NF-KB signaling pathway may be of great importance in removing CSCs and in cancer management.

Considering the facts of signaling mechanisms in CSC cell proliferation and oncogenicity, it is very likely that targeting of deregulated signaling could be of great clinical relevance [18]. Though enormous efforts have been made to identify and target aberrant signaling molecules and regulators of CSCs associated with human malignancies, still, a lot needs to be done to completely eradicate CSCs and to improve the overall disease-free survival.

## 4. Role of miRNA and Oncogenic Signaling Pathways in Human Cancer Stem Cells

MicroRNAs (miRNAs) are a family of endogenous, small (20–24 nucleotides), noncoding RNAs first identified in 1993 in *Caenorhabditis elegans*. Now, it has become evident that miRNAs are integral noncoding genetic components critically involved in the regulation of almost all the biological phenomena in various species, including vertebrates, generally through posttranslational modifications [36]. Increasing scientific reports demonstrate that usually miRNA regulates the expression of genes associated with various biological phenomenon such as homeostasis, development, proliferation, differentiation, and apoptosis, etc., through different feedback mechanisms, including binding to the open reading frame or to the 3′ untranslated regions (3′UTRs) of target genes causing a repression in translating mRNA or leading to mRNA degradation through activation of Argonaute (AGO) proteins which target the 3′ untranslated region [37,38,39,40]. A single miRNA can regulate the expression of multiple genes, and interestingly, expression of a gene can be modulated by a number of miRNAs. Further, it has been observed that around 60 percent of human genome functioning is governed by miRNAs related to most of the fundamental biological processes [41,42]. In general, biogenesis of miRNAs involves the transcription of introns and the long noncoding part of miRNA gene into primary miRNA by RNA polymerase II, and finally, after different steps of processing, functional miRNA is generated. Usually, the expression of miRNAs occurs in a tissue-specific fashion, which is regulated at the transcription level, and RNA polymerase II is critical in the transcription of miRNAs. Initially, in the nucleus, cleavage of the primary miRNA transcript through a complex composed of Drosha and a DGCR8 microprocessor complex subunit (DGCR8) results in the precursor miRNA [43]. Further processing of the primary or precursor miRNA in the cytoplasm through the coordinated action of various regulatory proteins such as RNase Dicer, AGO2 (Argonaute 2), and TRBP (trans-activation-responsive RNA-binding protein) formation of mature miRNA occurs. AGO protein, which is critical in the functioning of mature miRNAs, has four domains: (N)-terminal domain, the middle (MID) domain, the Piwi–Argonaute–Zwille (PAZ) domain, and the P-element-induced wimpy testes (PIWI) domains. Further, two other domains, L1 and L2, act as linkers also associated with the AGO protein [37,44].

Deregulated expression and signaling of miRNA have been well-studied in the pathogenesis of most human diseases including cancer [45,46]. Aberrant expression of miRNA often due to genetic modifications (genetic and epigenetic) is vital for the initiation and progression of human malignancies as they act as both tumor suppressors and oncogenes [46]. A number of investigations demonstrated the underlying oncogenic potential of miRNAs that includes targeted mRNA degradation at the 3′-untranslated region (3′-UTR) of several genes associated with the regulation of normal cellular homeostasis such as cell proliferation, cell cycle regulation, differentiation, development, migration, angiogenesis, and apoptosis [47,48].

**Figure 2:**
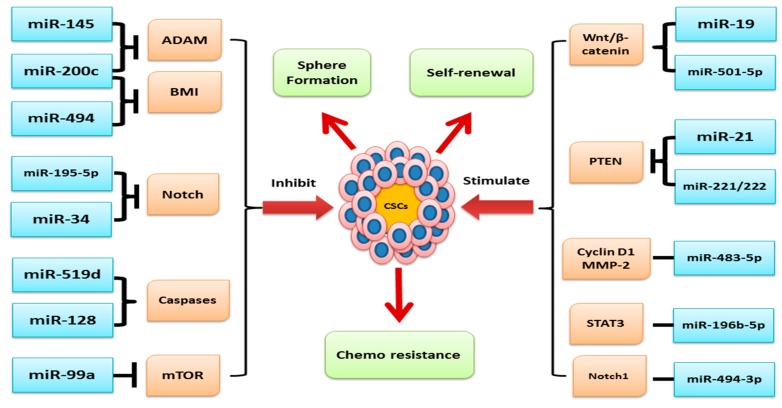
miRNAs in regulating cancer stem cells characteristics: miRNAs can both inhibit or stimulate the development and characteristic of CSCs such as the ability of self-renewal, sphere formation, and chemoresistance through targeting signaling pathways involved in their survival and proliferation. Many miRNAs were found to inhibit CSCs: miR-145 and miR-200c through inhibiting ADAM, miR-494 via BMI, miR-195-5p and miR-34 via inhibiting the Notch1 pathway, and miR-99a through inhibiting mammalian target of rapamycin (mTOR). Conversely, miR-519d and miR-128 inhibit CSCs via activating caspases. On the other hand, miRNAs can stimulate CSCs development: miR-19 and miR-501-5p through activating the wingless (WNT)/β-catenin signaling pathway; miR-21 and miR-221/222 via inhibiting phosphatase and tensin homolog (PTEN); and miR-483-5p, miR-196b-5p, and miR-494-3p through activating the Cyclin D1, STAT3, and Notch1 signaling pathways, respectively.

miRNAs play a critical role in human cancer initiation, progression, and prognosis. Chromosomal sites coding for oncogenic miRNAs which are associated with negative regulation of tumor repressors genes can be amplified in relation to the development of cancer, leading to oncogenic miRNAs upregulation and silencing of the tumor repressors genes. Conversely, miRNAs that target oncogenes are usually located in a fragile region where mutations or deletion can occur, causing a decrease or loss of miRNAs, hence overexpressing their target oncogenes [39]. A study reported that about 50% of miRNAs targeting oncogenes are positioned in fragile sites and are involved in cancer progression [49]. Various findings revealed that low and high expression of miRNA function as tumor suppressors and oncogenes, respectively, in human malignancies [50] (Figure 2). Furthermore, abnormal expression of miRNAs impacts the processes involved in the progression of cancer, such as promoting tissue invasion, metastasis, drug resistance, and stimulating antiapoptotic activity. Recent studies show that miRNAs are associated with tumor initiation and development through regulating CSC characteristics including the ability of self-renewal, drug resistance, and tumorigenicity [39]. Interestingly, miRNAs can modulate/deregulate the expression and functioning of genes and their products associated with the signaling pathways which are integral for the maintenance, growth, and function of CSCs such as Wnt/β-catenin, Notch, and others [50]. It has now become evident that miRNAs play a vital role in various developmental and regulatory mechanisms of stem cells such as maintenance, reprogramming, pluripotency, and differentiation by targeting various differentiation pathways, and thus, the overall regulatory action of miRNAs on stem cells suggests a critical role of miRNAs in the development of mammals [2,51]. 

Initially, it was the discovery of let-7 and lin-4 miRNA in *Caenorhabditis elegans* which revealed that miRNAs play a significant role in regulating the various molecular mechanisms related to the embryonic development and function of stem cells in mammals. Further, earlier reports have shown the crucial presence of miRNA for the development and functioning of stem cells such as the depletion of Dicer1, an enzyme crucial for miRNA biogenesis inhibits embryonic stem cell formation and lethality in mouse [52,53]. *DGCR8* is a gene essential to the development of stemness features, and knocking it out in mouse embryonic stem cells results in the altered expression of the markers of stemness such as *Rex1*, Oct4, *Nanog*, and *Sox2* and causes deregulation of cell cycles and differentiation [54,55]. The family of Let-7 is another important regulator for the differentiation of ESC. ESCs lack members of the mature let-7 family, and they accumulate only upon their differentiation. Let-7c rescued DGCR8^−/−^ differentiation defects through downregulating *Nanog*, *Sox2*, and *Oct4*. Family members of miR-34 are directly targeted by p53 and function as tumor repressors to inhibit reprogramming via pluripotency gene suppression including N-myc, Sox2, and Nanog. Since the evidence showed that members of the miR-34 and let-7 families are tumor repressor miRNAs, this suggests that miRNAs are critical players in carcinogenesis and progression via targeting CSCs [39]. 

An increasing number of studies have shown the oncogenic potential or role of miRNA in carcinogenesis which acts both as a tumor suppressor and tumorigenic. For instance, upregulation of miR-200 in breast cancer stem cells (BCSCs) disturbs the formation of the colonies in vitro and suppresses in vivo tumorigenesis. Furthermore, family members of miR-34 are activated via p53 and facilitate cell-cycle arrest and apoptosis. Moreover, oncogenic HMGA2 and RAS oncoproteins are targeted by let-7 miRNAs. In several types of cancer, reduced expression of miR-34 and let-7 trigger oncogenesis and upregulation causes suppression of tumor growth. However, members of the miR-181 and miR-155 families have been reported as oncogenic miRNAs which can stimulate self-renewal ability, colony formation, and development of tumor in breast cancer. In prostate cancer, reduced expression of several miRNAs such as miR-34a, let-7b, miR-106a, and miR-141 has been detected in CSCs, whereas some other miRNAs were found to be highly expressed, like miR-301 and miR-452. In addition, miRNAs profiling in breast CSCs showed that expression of miR-203 and miR-375 were markedly downregulated, whereas miR-100, miR-221, miR-222, and miR-125b expression were significantly upregulated [7].

Carcinogenesis or the development of cancer is a highly complex and heterogenic process orchestrated by a number of deregulated signal transduction pathways such as PI3K/Akt; Wnt/β-catenin; Notch; bone morphogenetic protein (BMP)/TGF-β; and Hedgehog, JAK/STAT, and NF-kB, etc. Generally, these signaling pathways play a central role in development and in survival processes of the multicellular organisms via regulation of the functioning of stem cells in response to environmental and biological stimuli. An increasing number of findings revealed that miRNAs play a very important role in ensuring the activation and functioning of the abovementioned signaling pathways [56,57]. Dysregulated expression of miRNAs often distorts the normal functioning of the abovementioned signaling machinery associated with human cancer pathogenesis [57]. Here, we have elaborated the role of miRNAs in regulating the CSC features in the pathogenesis of various human malignancies (Table 1).

**Figure 3:**
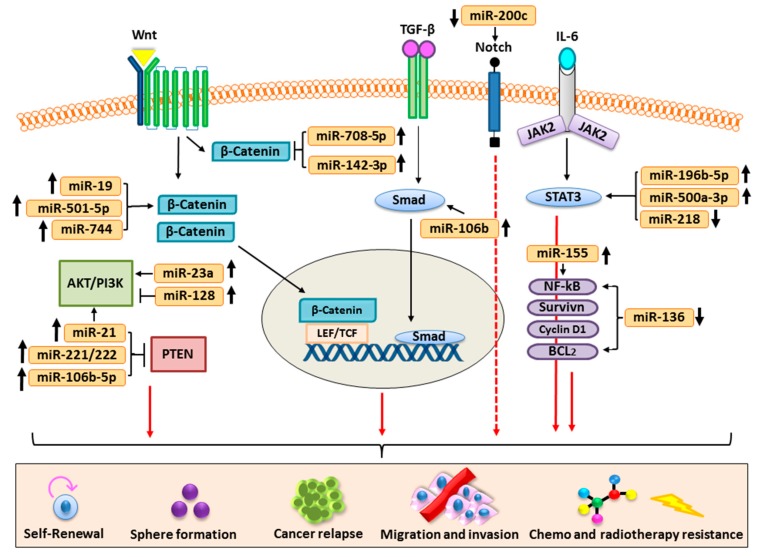
Mechanistic overview of miRNA-mediated regulation of the stemness of cancer stem cells via targeting signaling pathways: miRNAs can act as both suppressive and enhancer of stem cells features such as self-renewal, sphere formation, cancer relapse, migration, invasion, and chemo- and radiotherapy resistance. Via targeting the Wnt/β-catenin signaling pathway, many miRNAs regulate the stem cell features by targeting β-catenin. Upregulation of miR-19, miR-501-5p, and miR-744 stimulates the activation of β-catenin. Hence, it is translocated to the nucleus and stimulates gene expression. Conversely, upregulation of miR-708-5p and miR-142-3p inhibits β-catenin activation and prevents its accumulation in the cytoplasm. Another signaling pathway targeted by miRNAs is AKT/PI3K/PTEN. MiR-21, miR-221/222, and miR-106b-5p upregulation inhibit the function of PTEN. Furthermore, upregulation of miR-21 and miR-23a stimulate the activation of the AKT/PI3K pathway, whereas upregulation of miR-128 inhibits its activation. Upregulation of miR-106b is found to act on TGF-β through targeting Smad. Downregulation of miR-200c stimulates the activation of Notch pathways and, hence, enhances CSC features. Another important signaling pathway associated with CSC development is the JAK/STAT pathway. Upregulation of miR-196b-5p and miR-500a-3p and downregulation of miR-218 stimulate the activation of STAT3 molecule. Further, downregulation of miR-136 stimulates CSCs as a result of activating various proteins including *NF-kB*, *survivin*, *cyclin D1*, and *Bcl2*. In addition, upregulation of miR-155 activates NF-kB. Taken together, the stem cell features can be stimulated or suppressed by miRNA-regulated expression of signaling proteins.

Further, we have also elaborated how miRNAs modulate signaling pathways associated with CSCs and highlighted that exclusive targeting of the dysregulated miRNAs can be of great therapeutic significance in human cancers (Figure 3).

### 4.1. Colorectal Cancer

Colorectal cancer (CRC) is the third most commonly diagnosed malignancy and the fourth leading cause of cancer death in the world, accounting for more than 1.4 million new cases and around one million deaths annually [125,126]. Available therapeutic measures such as surgery and chemo- and radiotherapy for CRC are not adequate, and the presence of CSCs has been considered as one of the major underlying reasons [62]. Various findings suggest that deregulated expression and functioning of miRNAs are the main effector molecules associated with maintenance growth and functioning of colorectal CSCs [127].

In colorectal CSCs, miRNAs have been reported to be involved in regulating many signaling pathways. miR-21 is the most studied miRNA in colonic CSCs. Upregulation of miR-21 is reported in CSCs of various colorectal cancer cell lines including HT-29 and HCT-116 [128]. miR-21 overexpression in colon cancer cells is critically associated with increased colon CSCs and enhanced stemness features. miR-21 along with its target gene *PTEN* regulates the colon CSCs functioning. Roy et al. demonstrated that expression of miR-21 is increased many folds in colon CSCs as compared to their parental cells. Furthermore, the expression levels of *PTEN* decreased in the colon sphere because of miR-21-elevated expression. Consequently, AKT signaling pathway is activated and it is thought to have a critical role in colonic CSC regulation of tumorigenic properties [58]. 

Chemoresistance in CRC is one of the critical challenges of cancer therapy, and CSCs have been shown to play an important role in this phenomenon as demonstrated by an array of reports. miR-215 has been shown to play a critical role in CRC chemoresistance by reducing DTL expression-mediated G2-arrest and inhibition of cell proliferation [59]. miR-148a is another important tumor suppressor miRNA that has been shown to play a vital role in the regulation of CRC cell proliferation and invasion. Recently, it has been demonstrated that reduced expression of miR-148a in cisplatin-resistant CRC cells SW480 causes enhanced stem cell marker expression via targeting the Wnt/β-catenin signaling pathways. Further, overexpression of miR-148a results in reduced stemness features, increased chemosensitivity to cisplatin, and apoptosis in SW480 cells, suggesting the critical role of miR-148a in regulating CRC-related CSCs associated with chemoresistance [60]. Another investigation by Chen et al. revealed that overexpression of miR-199a/b causes chemotherapeutic drug resistance via targeting glycogen synthase kinase 3 β (Gsk3β)-mediated modulation of the Wnt/β-catenin-ABCG2 signaling pathway in colorectal cancer stem cells [61].

In this context, the role of STAT3 and miRNA in chemoresistance, particularly miR-196b-5p which is often deregulated in CRC patients, is well elucidated. One of the findings revealed that miR-196b-5p plays a central role in the maintenance of CSCs traits associated with chemoresistance to cancer therapeutic drugs via targeting STAT3 signaling pathway in CRC stem cells [62].

Now, it is well elucidated that miRNAs are critically associated with dysregulation of multiple crucial pathways in CRC. miR-31 has emerged as a potential driver for the colon oncogenesis via targeting EphB2 and EphA2 signaling pathways associated with the regulation of stemness, differentiation, tumor heterogeneity, and poor therapeutic outcomes in CRC patients [63].

The oncogenic role of dysregulated miR-27a has been well elucidated in a number of human malignancies including CRC via enhanced cell proliferation, invasion and migration, and EMT. In one of the recent studies, it has been found that overexpression of miR-27a in CRC stem cells which is associated with resistance as knocking down of miR-27a sensitizes CRC stem cells towards apoptosis via activation of the Apaf-1/caspase-9 apoptosome-mediated apoptotic pathway [64].

Deregulated expression and functioning of miR-372/373 which are critically related to the growth and functioning of stem cells have been observed in various cancer types including CRC. Wang et al. used The Cancer Genome Atlas data and characterized the upregulated expression of miR-372/373 in CRC tissues. They demonstrated that the upregulated expression of miR-372/373 enhanced CSC features via enrichment of stem-cell-positive cells ultimately responsible for chemoresistance, self-renewal, and metastasis of CRCs. Further, they also elucidated the underlying mechanism of miR-372/373-induced stemness and revealed that activated Nanog and Hedgehog signaling pathways are critically associated with the development and function of stem cells while the functioning of NFκB, mitogen-activated protein kinase (MAPK)/Erk, and VDR signaling mechanisms vital for differentiation was inhibited by miR-372/373, which suggests that miR-372/373 upregulates CRC stemness features via acting on various signaling mechanisms associated with the regulation of stemness and differentiation [65].

Self-renewal potential of cancer stem cells is a feature which has huge impact on oncogenicity as it poses great therapeutic challenges, and it has been observed that miRNAs are critical in the self-renewal feature of CSCs via modulating expression and functioning of the signaling molecules related to a number of pathways such as JAK/STAT, TGFβ, PI3K/Akt, and MAPK [66]. Generally, surface markers such as CD133, CD44, LGR5, and ALDH1 present in colon CSCs are often shared by normal colon stem cells, thereby limiting the potential of targeted therapy. Doublecortin-like kinase 1 (DCLK1), a novel stem cell marker specific to tumor cells, has been identified. Interestingly, expression of DCLK1 has been widely detected in clinical colon cancer specimen while DCLK1-positive cells in normal colon tissue are very rare. Recently it has been found that miR-137 acts as a tumor suppressor in colorectal cancer via suppression of uncontrolled cell proliferation by DCLK1 downregulation [67].

Deregulation of asymmetrical cell division (ACD) is often detected in many forms of cancer, including CRC, and play a vital role in tumor growth. An investigation by Hwang et al. showed suppressed ACD in colon CSCs and found that miR-146a plays an important role in the dysregulation of ACD through β-catenin [68]. It has been observed that the STAT3 signaling pathway is activated by miR-196b-5p in CRC. miR-196b-5p overexpression led to an increase in the nuclear STAT3 level, whereas miR-196b-5p silencing decreased STAT3 nuclear expression along with its downstream target genes, including *BIRC5*, *BCL2*, and *BCL-XL*. Upregulation of MiR-196b-5p-enhanced colorectal cancer stem cells (CRCSCs) spheroids formation, whereas its silencing causes inhibition of the spheroid formation. Moreover, upregulation of miR-196b-5p induced stem cells markers such as NANOG, SOX2, and OCT4, while its silencing repressed the expression of these markers, suggesting that miR-196b-5p upregulates CSC features in CRC via STAT3 deregulation [62].

Further another investigation by Jin et al. revealed the effect of miR-195-5p on maintaining CRCSCs as they found that expression of SOX2 and CD133 stem-cell markers are inhibited by miR-195-5p in the SW620 colon cancer cell line, while miR-195-5p silencing stimulated SOX2 and CD133 expression in the HT29 cell line, suggesting that miR-195-5p inhibits stemness features of colorectal CSCs. Further, they also treated HT-29 and SW620 with miR-195-5p, followed by treatment with 5-FU, and found that miR-195-5p repressed cell viability by enhanced apoptosis. MiR-195-5p reduced the rate of tumorsphere formation, whereas the rate increased after using anti-miR-195-5p, suggesting that miR-195-5p sensitized colorectal cancer cells resistant to chemotherapeutic drugs as it regulates the expression and functioning of *RBPJ* and *Notch2*, which are well-known genes associated with chemoresistance and colorectal CSCs. Ectopic expression of miR-195-5p in the SW620 cell line reduced the expression of *RBPJ* and *Notch2* while using the miR-195-5p inhibitor in the HT-29 cell line increased their expression [69].

### 4.2. Lung Cancer

Lung cancer is one of the prime causes of morbidity and mortality related to cancer. Around 1.8 million cases are reported annually, and 1.6 million people die every year because of lung cancer [129]. Lung cancer is divided into two categories: non-small cell lung cancers (NSCLC), which accounts for about 80% of reported cases, and small cell lung cancers (SCLC), which accounts for 20% of all lung cancer cases. Dysregulated expression of miRNA has been shown to play a critical role in the regulatory mechanisms associated with the initiation and progression of lung cancer as they target genes related to various processes such as apoptosis, proliferation, inflammatory response, cell cycle regulation, stress responses, lung CSC maintenance, invasion, migration, differentiation, and development [130]. The increasing number of findings reveals that miRNAs are the central regulatory molecules for the maintenance of lung CSCs associated with metastasis, drug resistance, and tumor self-renewal by effectively controlling various signaling pathways associated with cell cycles, proliferation, apoptosis, immune response, and differentiation of lung CSCs [131,132].

Several reports on the role of miRNAs in regulating lung CSCs associated with a number of challenges related to cancer management such as self-renewal, disease recurrence, resistance, and metastasis have been published. For instance, miR-128, a well-known tumor suppressor often suppressed/deregulated in different human cancers including lung cancer, plays an important role in the self-renewal of CSCs and resistance via targeting AKT/ERK signaling pathways [72]. Further, downregulation of miR-128 in lung cancer patients is associated with tumor differentiation, pathological changes, and metastasis by targeting ERK, AKT, and p38 signaling pathways [73]. It has been shown that in the gefitinib-resistant lung cancer PC9-CSCs, expression of miR-128 was markedly suppressed as compared to non-CSCs and upregulation of miR-128 reverses the gefitinib resistance of the lung cancer stem cells by inhibiting the c-met/PI3K/AKT pathway [72]. In another study, it was found that BRM270, an extract from seven plants, triggers the expression of miR-128 in lung CSCs and sensitized chemoresistant A549 lung cancer cells by attenuating lung CSC traits via inhibition of the activation of VEGF/PI3K/AKT signaling pathways [70]. Downregulation of miR-218 in lung cancer cells has been shown to induce constitutive activation of STAT3, which is critically associated with oncogenesis. In ALDH-positive lung CSCs, dysregulated expression of miR-218 upregulated IL-6/JAK-STAT3 signaling and cancer stemness features [71].

The ectopic miR-181b expression has been shown to inhibited lung CSCs stemness and sensitized cisplatin-resistant cancer cells in addition to the reduction in tumor size via targeting the Notch2 signaling pathway [74]. TGFβ has a pivotal mechanism for the generation of CSCs maintenance and proliferation through induction of epithelial–mesenchymal transition. miR-138 is a vital target for TGFβ-induced EMT in primary lung cancer cells [75].

Another recent investigation by Yang et. al. revealed that miR-5100 is upregulated in both lung CSCs and non-lung CSCs and has been found as a prime cause for cisplatin resistance. They reported that miR-5100 expression was more in CD44^+^ CD133^+^ lung CSCs than in non-CSCs and was associated with enhanced stemness features and tumorigenicity. Interestingly, they further reported that downregulation of miR-5100 attenuated stemness features and chemotherapeutic resistance to cisplatin via inhibiting Rab6 through the mitochondrial apoptosis pathway. Deregulated expression of c-MYC, a crucial regulator of CSCs and its association with chemoresistance has been reported in various human malignancies. Interestingly miR-214-mediated inhibition of c-MYC signaling has been to suppress CSC stem-like features and cisplatin resistance in lung cancer cells [76].

Dysregulation of miR-708-5p expression and the associated signaling has been one of the driving forces for lung cancer stemness and its associated pathogenesis. It has been observed that upregulation of miR-708-5p expression suppresses lung cancer stemness, epigenetic changes, and tumorigenesis via repressing Wnt/β-catenin signaling [77]. Recently, it has been found that miR-873 or miR-125a-3p inhibited HuR-induced upregulation in the stemness markers (Oct4, Nanog, and ALDH) of lung CSCs [78].

Another important challenge of lung cancer therapy is the role of deregulated functioning of miR-23a and stem cells in making the lung cancer cells resistant to epidermal growth factor receptor-targeted tyrosine kinase inhibitors (EGFR-TKIs), commonly used for lung cancer treatment. It has been demonstrated that the uncontrolled upregulation of miR-23a in lung CSCs associated with erlotinib resistance and inhibition of miR-23a accelerated the anticancer effect of erlotinib via modulating the functioning of PTEN/PI3K/AKT pathways [79].

Faversani et al. reported that overexpression of miR-494-3p in A549 lung cancer cells enhances the cancer cell stemness and proliferation via modulation of Notch1/PI3K signaling associated with the development and pathogenies of lung cancers [80]. miRNAs such as miR-19a and miR-19b induced CSC maintenance, and oncogenesis involved targeting of Wnt/β-catenin signaling cascade in addition to others. The upregulated expression of miR-19a/19b in lung CSCs play a major role in the maintenance of stemness features and tumorigenesis via Wnt/β-catenin pathway activation. Sulforaphane, a natural compound, has been shown to inhibit lung CSC-induced tumor formation via downregulation of miR-19a- and miR-19b-mediated suppression of Wnt/β-catenin activation [81]. Another important finding revealed that the role of the TIC or cancer stem cells in the stemness and functional heterogeneity of tumors is due to the presence of the CSCs-specific miRNAs, miR-1246 and miR-1290, which are critical for the tumor initiation and progression or pathogenesis of human lung cancer. Further, loss or the inhibition of either of the miRNAs suppressed the carcinogenic and/or metastatic potential of CSCs [133].

### 4.3. Breast Cancer

Breast cancer is the most common type of heterogeneous disease-related mortality in woman around the world. BCSCs are critical for tumor formation, drawbacks associated with therapy, and metastasis. Increasing evidences suggest that miRNAs are the central regulatory molecules for growth and proliferation of breast cancer cells and BCSCs as they regulate the expression and functioning of genes associated with a series of signaling pathways critical for normal cellular homeostasis and pathogenesis of various human diseases including breast cancer [134].

Recently Wu et al. demonstrated that miR-29a is upregulated in BCSCs, MCF-7, and breast cancer tissues. They elucidated that basic fibroblast growth factor (bFGF)-induced upregulation of miR-29a markedly enhanced the migration and metastasis of BCSCs and other aggressive breast cancer cells by suppressing the expression of SUV420H2-mediated downregulation of H4K20me3 [135]. Another elucidation related to the explanation of which and how miRNAs are important determents in triple negative breast cancer (TNBC) pathogenesis found that the downregulated expression of miR-1287-5p both in mammospheres (BCSCs) and human breast cancer tissue is critical for poor prognosis and survival via modulation of PI3Kinase signaling cascade. Further, it has been validated that the upregulation of miR-1287-5p inhibits cell proliferation and induces cell cycle arrest, apoptosis, and tumor formation in triple negative breast cancer via modulation of PI3Kinase signaling pathway [82].

BCSCs exhibit upregulated miR-137 that regulates the expression of *FSTL1*, a gene often associated with the deregulation of signaling mechanisms in various human disorders including cancer and associated with stemness maintenance and enhanced chemoresistance via targeting β3/Wnt signaling pathway [83]. Another investigation revealed that B-cell lymphoma/leukemia 11A (*BCL11A*), a zinc-finger transcription factor, is upregulated in TNBC stem cells and tissues and enhanced BCSCs stemness. miR-137, often lowly expressed in TNBC and BCSCs, has been shown to downregulate BCSC stemness potentials and in vivo tumorigenesis via targeting *BCL11A* downregulation [84].

Recently the role of tumor suppressor miR-34a has been investigated which controls the BCSC stemness features including self-renewal and normal mammary physiology by targeting Wnt/β-catenin signaling, which is critically associated with growth and maintenance of stem cells both in normal and cancer tissue of the mammary gland. Further, it has also been validated that miR-34a has potential to limit BCSC pools. Hence, targeting of the upregulation of miR-34a-dependent signaling pathways can be an ideal approach for the eradication of the BCSCs [85]. miR-34a has been shown to inhibit the insulin-like growth factor II (IGFII) mRNA binding protein (IMP3)-induced stemness of BCSCs and tumorigenesis in TNBC cells and tissues [136]. Another study by Lin et al. [137] demonstrate that nanoformulation of miR-34a successfully removes BCSCs via targeting C22ORF28 (chromosome 22 open reading frame 28), which act as a direct and functional target of TV-miR-34a. By using hTERT promoter-driven VISA delivery of miR-34a plasmid, they further showed TV-miR-34a markedly suppressed tumor-initiating properties of BCSC in vitro and in vivo and also reported that TV-miR-34a plasmid synergizes with docetaxel for eradicating BCSC by targeting C22ORF28 [96].

miR-628, another important miRNA deregulated in human cancer pathogenesis, has been shown to suppresses BCSC stemness features by targeting Ras/Rac guanine nucleotide exchange factor 1 (SOS1) [138]. Interestingly, hypoxia or hypoxic tumor microenvironments or tumor vicinity has been well-known as a tumorigenic stimulus via targeting a number of biological mechanisms including miRNAs. A recent finding shows that hypoxic tumor environments enhanced BCSC stem-like properties associated with the available therapeutic drawbacks via targeting deregulated increased in miR-210 [139]. An investigation on the human breast cancer sample and BCSC spheroids have revealed that deregulated expression of two miRNAs, miR-9 and miR-221, have strong tumorigenic potential as they enhanced BCSC properties related to cancer development and recurrence via targeting of a number of genes involved with carcinogenesis [140].

Recently, it has been observed that miR-142-3p suppressed cancer stem-cell characteristics and radioresistance by targeting β-catenin pathway [141]. Furthermore, miR-27a is known to sensitize TNBC cells towards radiation therapy by targeting CDC27 [142]. Deregulated expression of miRNA-140-5p, well-known for the critical role in cancer development, was detected in preoperative breast cancer patients at various stages and regulates BCSC expression by modulating various signaling pathways such as Wnt, SOX2, and SOX9 [86]. Further, miR-140-5p has been shown to suppress the growth and sphere-forming capacities of BCSCs via inhibiting the Wnt/β-catenin pathway and enhanced chemosensitivity in BCSCs to doxorubicin [143]. Dysregulated expression of miRNAs and associated signaling in TNBC-associated BCSCs (TNBC SC) is critical for carcinogenesis. One of the findings revealed that 112 microRNAs were upregulated and 81 were downregulated. Moreover, 13 novel miRNAs were identified and many of these were related to regulating TNBC malignancies, such as BCSC self-renewal, tumorsphere formation, and metastasis. Furthermore, downregulation of miR-4319 was critical for the TNBC SC capacity of self-renewal, tumor formation, and enhanced metastases, while overexpression of miR-4319 markedly inhibits the tumorigenicity of stem cells in TNBC by modulation of E2F2 expression. It has been further revealed that E2F2, a member of the E2F family of transcription factors, plays a vital role in cell proliferation, that transformation, embryonic development, and differentiation is vital for stem cells self-renewal, and that miR-4319 reduces the stemness and tumorigenicity of TNBC SC by inhibiting the expression of E2F2 [144]. Another investigation shows the downregulated expression of miR-130a-3p in human breast cancer tissues and exosomes from circulating blood. Further, they found that upregulation of miR-130a-3p in BCSCs inhibited carcinogenesis and knockdown of miR-130a-3p enhanced the carcinogenic features of BCSCs by modulating the expression and functioning of RAB5B, a member of the RAS oncogene family [145].

miRNA Let-7 has been shown to potentiate the effectiveness of endocrine therapy via regulating the stemness features of BCSCs. It also enhances the anticancer potential of tamoxifen via suppressing growth and stemness of BCSCs and associated tumorigenesis via targeting the Wnt signaling pathways [146]. miR-155 is an oncogenic molecule often dysregulated in most human malignancies including breast cancer patient samples as well as in cell lines. Various experimental approaches showed that inhibition of miR-155 markedly inhibits proliferation of breast cancer cells. Furthermore, downregulation of miR-155 reduces stemness markers of BCSCs such as ABCG2, CD44, and CD90 and sensitizes TNBC cells to doxorubicinol [147]. miR-205, which has both oncogenic and tumor suppressive actions, has been shown to play a vital role in the maintenance of stemness features of TNBC stem cells in SUM159PT as it inhibits the renewal of BCSC stemness most likely through modulating STAT3 signaling pathways [87].

miR-31 is highly expressed in mammary stem cells, promotes the proliferation of mammary epithelial cells, and enhances the expansion of mammary stem cells. Further, inhibition of miR-31 in MMTV-PyVT mice lead to reduce tumor growth, decrease in number of BCSCs, and reduced tumor-initiating potential and metastasis by modulating a number of signaling pathways, including Prlr/Stat5, TGFβ and Wnt/β-catenin, suggesting that miR-31 is critical for BCSC stemness and associated tumorigenesis [88].

miR-221/222 is critical for the growth and maintenance of BCSC as they are highly overexpressed in MDA-MB-231 cells by targeting PTEN signaling. Interestingly, ectopic expression of miR-221/222 and PTEN inhibition enhanced BCSC enrichment and tumor growth via targeting PTEN which in turn activates Akt/NF-κB/COX-2 [89]. miR-519d critically associated with breast carcinogenesis via modulating survival pathways. Recently, reduced expression of miR-519d was detected in BCSCs, and induced upregulated expression of miR-519d in cancer stem cells has been shown to sensitize towards chemotherapeutic drug cisplatin through activation of the mitochondrial apoptotic pathway. As a result, this significantly increased their sensitivity to cisplatin through *MCL-1*, a member of the proapoptotic Bcl-2 family, a dependent mitochondrial pathway in BCSCs [90]. In addition to abovementioned reports, there are a number of findings showing the dysregulated expression and signaling of miRNAs in the maintenance of BCSCs and carcinogenesis-like induction of pro-inflammatory responses in BCSCs and stromal cells [148], in the regulation of BCSC differentiation and fate by miR-600 through Wnt Signaling [149], and in the inhibition of BCSCs stemness and chemotherapeutic drug tamoxifen resistance in human ER-positive breast cancer by miR-375 through degradation of *HOXB3* [150].

### 4.4. Gastric Cancer

Gastric cancer (GC) is the fourth most common type of cancer ranked as the third leading cause of cancer-related death worldwide predominately in East Asian countries such as China [151]. The development of GC is a complex and multistep process among the host, environment, and microorganisms leading to dysregulation of genetic and epigenetic signaling mechanisms [152]. The multidisciplinary therapeutic approach involving gastrectomy, chemotherapy, and radiation therapy is the primary treatment strategy, with a survival rate ranging between 25 to 30 percent, and poor prognosis in the patients with late stage of GC due to tumor relapse and metastasis [153]. However, the development of tumor resistance towards available therapeutic options is the major clinical concern. The increasing number of studies suggests that gastric cancer stem cells (GCSCs) are the prime cause of tumor formation, maintenance and metastasis, tumor relapse, and drug resistance and the miRNAs are the main effector molecules regulating stemness of GCSCs [152,154]. Recently, upregulated miR-135b expression was observed in mice with GC cells and organoids due to enhanced pro-inflammatory cytokine interleukin-1 and promoted stemness and metastatic potential of GC cells by targeting FOXN3 and RECKS expression [155].

miR-26a has been shown to target HOXC9, which has strong oncogenic potential under deregulated conditions. Deregulated expression of HOXC9 in GC tissues has been linked with poor prognosis and also enhances GCSCs and metastasis. Interestingly, miR-26a has been shown to suppress HOXC9 and inhibits its oncogenic potential and stemness features of self-renewal, suggesting that miR-26a-mediated HOXC9 silencing could be viable for GC therapy [91]. Further, miRNA-19b/20a/92a have been shown to enhance stemness features like self-renewal of GCSCs by acting on E2F1 and HIPK1 via activation of β-catenin signaling mechanisms [92]. In GC cell lines NCI-N87 cells, deregulation of miR-206 has been linked with GCSC maintenance, tumorigenesis, and drug resistance. Further, downregulation of miR-206 in GC enhances GCSCs and carcinogenesis, while its overexpression suppressed the formation of GCSCs and associated tumorigenesis via downregulation of ETS homologous factor (EHF), a member of the E26 transformation-specific (ETS) transcription factors, with oncogenic potential in gastric tissues and is often associated with poor clinical outcomes [156].

miR-483-5p, an important oncogenic molecule deregulated in various types of human malignancies, has the potential of a cancer biomarker. Overexpression of miR-483-5p in GCSCs has been reported to enhance cell proliferation, suppression of apoptosis, tumor cell invasion, and stemness features via activation of Wnt/β-catenin signaling-mediated upregulation of the antiapoptotic proteins and proteins associated with invasion and self-renewal of GCSCs [93]. The miR-106b family members often deregulated in various cancer types including GC and have been shown to target cell cycle regulators the cyclin-dependent kinases such as p21. In CD44(+) GCSCs, miR-106b enhances the stemness traits of GCSCs such as EMT, self-renewal, and invasion via modulation of the TGF-β/Smad signaling pathway, and further, it is found that knockdown of miR-106b inhibits activation of TGF-β/Smad signaling and reduced stemness features of GCSCs [94].

The miRNA miR-17-92 cluster, miR-19b, miR-20a, and miR-92a are the important oncogenic molecules as their deregulated functioning is associated with carcinogenesis and known to widely overexpress in normal, and CSCs has been shown to regulate self-renewal of GCSCs both in vitro and in vivo conditions by targeting E2F1 and HIPK1 and activation of Wnt–β-catenin signaling pathways [157]. The development of resistance towards chemotherapeutic drugs such as cisplatin is an important concern related to GC therapy, and it has been demonstrated that miR-132 plays a vital role in GC cisplatin resistance by modulating the SIRT1/CREB/ABCG2 signaling pathways [95]. miR-501-5p, another important molecule often overexpressed in cancer tissues and acts as the oncogenic driver, has been overexpressed both in cell lines, and GC patient samples are upregulated and enhance carcinogenesis and GCSCs stemness via CSC-like phenotype via dickkopf-1 (DKK1), NKD1, and GSK3β [96]. Targeting of miRNAs has been accepted as an effective mechanism for cancer management. One of the findings reported that Sulforaphane, a natural compound, suppressed stemness features and oncogenicity of GCSCs via targeting IL-6R/STAT3 signaling pathways [97].

### 4.5. Prostate Cancer

Prostate cancer (PCa) is one of the most common cancers in men and is associated with high mortality and mortality around the world both in developed and developing countries, with higher incidence and mortality in African Americans, and is also the second leading cause of death among men in the developed world due to cancer [158]. PCa development is a complex and heterogenous phenomenon, and the available therapeutic options such given radiation therapy, hormonal therapy, or chemotherapy are not promising in the patients with advanced and/or metastatic disease and are associated with various complications. Increasing evidences revealed that the presence of CSCs in prostate cancer tissue is one of the sole reasons for the available therapeutic clinical failure as they play a critical role in self-renewal, epithelial-mesenchymal transition, metastasis, and drug resistance [159]. The role of miRNAs in the PCa heterogeneity and aggressiveness is very well elucidated in various preclinical and clinical reports carried out throughout the world. Further, deregulated expression of miRNAs has been shown to play a critical role in the maintenance of prostate cancer stem cells (PCaSCs) associated with metastasis, drug resistance, and therapeutic failures [11]. Therefore, here, we have documented the recent reports on the regulatory role of miRNAs in PCaSCs and the underlying mechanisms. miR-218 has been shown to function as a tumor suppressor in various types of human cancer including PCas. However, the underlying molecular mechanism is not well elucidated, and one of the findings show that miR-218 was reduced in PCa and suppresses the migration, invasion, and EMT of stemness of PCaSCs via suppression of GLI family zinc finger 1 (Gli1) [160]. Deregulated overexpression of miR-218 detected in PCa specimens including spheroids has been shown to upregulate the expansion and stemness of PCaSCs and markers like OCT4, SOX2, NANOG, CD44, KLF4, c-MYC, and metalloproteases via targeting activation of Wnt signaling pathway by suppressing GSK3β and SFRP1 [98]. A number of reports suggest that natural products could be a viable source of potential anticancer drugs, and one of the natural compounds curcumin has been shown to suppress the growth of cancer cells and cancer stem cells. Treatment with curcumin in CD44+/CD133+ human prostate cancer stem cells (HuPCaSCs) isolated from two pathologically different human PCa cell lines, 22RV1 and DU145, inhibits growth, invasion, and tumorigenicity of both non-stem prostate cancer cells and HuPCaSCs via targeting cell cycle arrest [161].

Further, miR-141, a miR-200 family member, has been shown to downregulate in various PCaSC populations present in tumors derived from animals as well as from patients with primary tumors. Interestingly, forced overexpression of miR-141 in PCa cells suppressed stemness of PCaSCs including growth and proliferation of stem cells, invasion, tumor recurrence, and metastasis via targeting a cohort of prometastatic genes including CD44, EZH2, and Rho GTPases, suggesting that miR-141 may be a viable target for PCa therapy [162]. Though genetic alterations in molecules associated with UPS (ubiquitin-proteasome system) play a vital role in tumorigenesis, recently, it has been discovered that dysregulation of miR-424 expression also causes perturbations in UPS which is associated with degradation of oncogenic transcription factors in prostate cancers such as STAT3. Mechanistically, deregulated overexpression of miR-424 suppresses posttranslational silencing of the E3 ubiquitin ligase COP1, resulting into reduced STAT3 degradation and thus enhancing cancer initiation and stemness features in prostate epithelial cells and further knockdown of miR-424 revert COP1-mediated degradation of STAT3. Overexpression of miR-424 in PCa cells enhanced cell migration and invasion, expression of EMT markers, and formation of prostatospheres (PSs) with increased stemness features associated with cancer stem cell-like phenotypes. Interestingly, anti-miR-424 treatment inhibits PS formation, suggesting a significant depletion in stemness features and carcinogenesis and via targeting E3 ubiquitin ligase and COP1-mediated STAT3 activation [99].

PCaSCs are the prime force for the recurrence, metastasis, and resistance associated with PCa. miR-302/367 cluster, a potent pluripotency regulator, overexpressed in PCa and enhance sphere formation with increased stemness markers such as NANOG, SOX2, OCT4, KLF4, and BMI-1 expression and resistance towards therapy. Knockdown of miR-302/367 inhibits tumorigenesis via regulating the LATS2/YAP pathway, suggesting that modulation of LATS2/YAP is critical for prostate cancer metastasis and resistance and, hence, targeting of this signaling axis may be of a great cancer therapeutic potential [100]. Another study related to androgen deprivation therapy and development of resistant in PCa suggests that the underlying mechanism involves a constitutively activated intrinsic inflammatory signaling circuit composed of IκBα/NF-κB(p65), miR-196b-3p, Meis2, and PPP3CC, which regulate stemness-associated transcription factors that trigger the enhanced tumorigenicity of CRPC cells, and that interruption or knockdown of any molecule of IκBα/NF-κB(p65), miR-196b-3p, Meis2, and PPP3CC signaling circuits inhibits castration-resistant prostate cancer (CRPC) development [101].

Genetic mapping analysis suggests that a particular location on chromosome 8p, a critical genetic alteration related to PCa initiation and metastasis, is directly associated with the downregulation of miR-383-is, which plays a critical role in PCa development via regulating CD44, a ubiquitous marker of PCaSCs, and the forced upregulation of miR-383 inhibited tumor-initiating capacity of PCaSCs and metastasis, suggesting that the restoration of miR-383 at the chr8p22 region may be an effective therapeutic modality against PCa [163]. It has been observed that miR-199a-3p is underexpressed in CD44+ PCaSCs and induced upregulation of miR-199a-3p in PCaSCs and that primary cancer cells suppressed growth, proliferation, and cancer-initiating potential of PCaSCs and tumors via targeting CD44 directly in addition to various other signaling molecules associated with cancer development like c-MYC, cyclin D1 (CCND1), and EGFR, suggesting that the attenuation of deregulated loss of miR-199a-3p may be viable for PCa therapy [164]. It has been observed that the upregulation of Lin28A and Lin28B expression resulted in the downregulation of Let-7 miRNA, found to modulate PCaSC stemness and phenotypes. The expressions of Lin28A and Lin28B enhance PCa stemness and tumorigenesis via the downregulation of Let-7 miRNA, suggesting that the deregulation of Lin28A and Lin28B/Let-7 miRNA axes promotes the acquisition of tumorigenic and CSC-like properties in prostate tumors. Further, knockdown of Lin28A and Lin28B has been shown to markedly reduce the expression of CSC markers, like Nanog, Sox2, POU5F1, KLF4, and BMI-1, along with reduced PCaSCs, suggesting that Lin28A and Lin28B targeting could selectively remove CSCs form PCa [165]. Exposure to arsenic has been shown to transform prostate stem cells via deregulation of a number of miRNAs, and loss of miR-143 is critical in the arsenic-induced malignantly transformed human prostate cells. Interestingly, forced overexpression of miR-143 in the arsenic-transformed PCaSCs suppressed various features of carcinogenesis via modulating MMP-9 and MMP-2 expression, cell proliferation, resistance to apoptosis, and SC self-renewal potential via regulation of LIMK1 and cofilin. This suggests that miR-143 may act as a potential biomarker and therapeutic target for arsenic-induced prostate cancer [102]. Downregulation of the tumor suppressor miR-7 has been shown to specifically inhibit the growth of PCaSCs and associated carcinogenesis by inhibiting KLF4, a stemness-related critical factor. Conversely, restoration of miR-7 abrogates the cellular and molecular alterations associated with PCa development via targeting KLF4/PI3K/Akt/p21 signaling pathways. Furthermore, it has also been found that miR-7-mediated inhibition of stemness features of PCa have potential to be maintained for generations [103]. There are various other reports about the role of miRNA in the maintenance and functioning of PCaSCs in PCa, like the deregulated overexpression of miR-143 which plays a critical role in the differentiation of PCaSCs and tumorigenesis via targeting the fibronectin type III domain containing 3B (FNDC3B) [166]; miR-320 which inhibits stemness features of PCa cells by inhibiting the activation of Wnt/β-catenin signaling [167]; and miR-34a which inhibits PCaSC growth and stemness and migration by targeting CD44 [168].

### 4.6. Pancreatic Cancer

Pancreatic cancer, particularly the pancreatic ductal adenocarcinoma (PDAC), is one of deadliest human cancers, comprising about 90% of all pancreatic cancers, and is associated with mortality and a low survival rate below 5% despite the advancement in available therapeutic options [169]. Recently, it has been demonstrated that expression of miR-100 and miR-125b in PDAC by TGF-β signaling to target similar signaling pathways co-operate to promote stemness and associated carcinogenesis, including EMT, and further reported that knockdown or suppression of miR-125b or miR-100 compromise the potential of TGF-β to induce cell migration and carcinogenesis in PDAC [104]. PRDM14, a transcriptional regulator that maintains pluripotency in embryonic stem cells, is overexpressed in pancreatic cancer tissues, and the inhibition of PRDM14 reduces sphere formation, stemness, stem cell markers, tumorigenesis, and metastasis in mice by upregulation of different miRNAs such as miR-125a-3p. This suggests that PRDM14 suppresses cancer stem-like phenotypes, including liver metastasis, via miRNA regulation, and siRNA-based therapy targeting [170].

A number of investigations revealed that pancreatic cancer stem cells (PCSCs) are critical for the drawbacks associated with therapy such as drug resistance and the emergence of cancer aggressiveness and metastasis. Recently, the role of miRNAs associated with the targeting of signaling components related to the maintenance of cancer stemness, growth, differentiation, and proliferation of PCSCs has been revealed [171]. It has been reported that the enhanced expression of miR-30 family members like miR-30a, -30b, and -30c in the stemness of CD133+ PCSCs shows resistance to gemcitabine, an important anticancer drug for pancreatic cancer, and further depicts higher migratory and invasive features with increased mesenchymal phenotype markers [172]. The dysregulated Wnt/β-catenin signaling pathway has been the prime pathway associated with the stemness features of PCSCs and is regulated by miRNAs. Interestingly, the deregulated overexpression of miR-744 detected in human pancreatic cancer specimens and cells and often correlated with poor patient survival has been shown to enhance Wnt/β-catenin signaling via targeting the inhibitors or the negative regulators of Wnt/β-catenin signaling such as secreted frizzled-related protein 1 (SFRP1), GSK3β, and transducing-like enhancer of split 3 (TLE3). Furthermore, the knockdown or inhibition of miR-744 downregulated stemness features of PCSCs and, hence, suggested that miR-744 is critical for stemness of pancreatic cancer cells and could act as a suitable prognostic biomarker and therapeutic target [173]. Another investigation showed aberrantly low expressions of miR-200c in PCSCs isolated from PANC-1 cells and is associated with the enhanced potential of proliferation, metastasis, and drug resistance while induced overexpression of miR-200c in PCSCs attenuated the stemness features [174]. Further, another mechanistic investigation reveals that the modulation of miR-200c expression inhibits the stemness of PCSCs via targeting Notch1 [105].

### 4.7. Hepatocellular Carcinoma (HCC)

Hepatocellular carcinoma (HCC) or liver cancer is one of the leading human cancer types that lead to high rates of mortality around the globe especially in Asian countries like China [175]. Despite considerable advancement in the therapeutics of HCC, increasing rates of cancer recurrence, resistance, and death are still the main challenges for HCC patient treatment and liver cancer stem cells (LCSCs) is one of the important factors for the failure of therapeutics and disease recurrence [176]. A number of reports show the critical role of miRNAs in the maintenance and functioning of LCSC-associated disease recurrence, metastasis, and drug resistance. Aberrantly high expressions of miR-6875-3p in HCC tissues and cell lines potentiates tumorigenesis and differentiation metastasis via upregulating the stemness features of LCSCs both in in vitro and in vivo experiments while knockdown of miR-6875-3p inhibits stemness of LCSCs and tumorigenesis via targeting the BTG2/FAK/Akt signaling pathway [106]. The Cancer Genome Atlas (TCGA)-based miRNA profiling of liver cancer samples and adjacent non-tumor tissues revealed many miRNAs, including miR-486, that were markedly downregulated in HCC tissues. Additionally, experiments on tumor spheres and liver cancer samples confirm the marked downregulation of miR-486 associated with LCSC stemness features while reduced expression of miR-486 repressed the self-renewal and invasion of CSCs in HCC development via targeting sirtuin 1 (Sirt1), which plays a critical role in the maintenance of the stemness and tumorigenic potential of LCSCs [177]. Recently, it has been shown that the expression of LCSC-specific miRNA miR-192-5p was found to be suppressed in a cohort of HCC patients. It was further noted that aberrant downregulation of miR-192-5p in HCC cells results in a marked increase in various types of CSC populations with stemness features via targeting PABPC4. In addition, they also reported that genetic (TP53 mutation) and epigenetic (miR-192 promoter hypermethylation) changes markedly contributed to the reduced level of miR-192-5p in multiple groups of CSCs + HCCs and HCCs, with a reduced level of miR-192-5p having shorter overall survival [178]. Increased expression of miR-106b-5p has been detected in HCC tissues and cell lines as compared to non-tumor tissues and hepatocytes which markedly enhanced the stemness features of LCSCs and metastasis via targeting PTEN through the PI3K/Akt pathway. Furthermore, it was found that the knockdown/inhibition of miR-106b-5p suppressed the stemness and tumorigenesis of LCSCs [107]. Chronic exposure to arsenite causes various types of human cancers including HCC by inducing the CSC stemness via inducing the aberrant expression and functioning of miRNAs. Arsenite-exposed liver cancer cells acquired stemness features by upregulation of miR-155 levels via activation of NF-κB. Further, spheroid formed due to arsenite exposure in liver cells showed enhanced stemness markers such as CD90, EpCAM, and OCT4, and interestingly, the inhibition of miR-155 suppressed spheroid formation by modulating QKI expression [179]. Another report revealed that exposure of human liver epithelial L-02 cells to arsenite causes upregulation of miR-191 which enhances epithelial-mesenchymal transition acquisition, spheroid formation, and stemness features while knockdown of miR-191 suppressed the stemness features of LCSCs via HIF-2α-mediated transcriptional activation [108].

CSCs potentiate tumor heterogeneity, stemness maintenance, carcinogenesis, and progression by modulating the expression and functioning of a number of genes both at the transcriptional and translational levels, and miRNAs are the major enforcing molecules. Overexpression of miR-217 often detected in HCC tissues promotes stemness features of LCSCs while knockdown of miR-217 inhibits stemness features and tumorigenesis in HCC. Mechanistically, miR-217 enhances the CSC-like phenotype via DKK1 targeting, which leads to activation of Wnt signaling, and this effect of miR-217 is reversed by the upregulation of DKK1 in miR-217 [109]. miR-500a-3p, another important miRNA, has been shown to trigger various types of human carcinogenesis including HCC. Enhanced expression of miR-500a-3p is often present in HCC patients and correlated with poor survival; it has been shown to promote stemness of LCSCs and tumorigenesis while its silencing abrogates stemness and tumorigenesis. Further, it has been shown that miR-500a-3p promotes the CSC features by targeting the negative regulators of SOCS2, SOCS4, and PTPN11 of STAT3, resulting in constitutive STAT3 activation as miR-500a-3p-induced stemness features reversed STAT3 inhibitors Stattic and S3I-201 in HCC cells [110]. Drug resistance due to CSCs is another important point due to CSCs. In HCC, it has been observed that upregulation of miR-137 reversed resistance and LCSC stemness features by degrading adenine nucleotide translocator 2 (ANT2) in hepatocellular carcinoma [180]. Expression of miR-589-5p and miR-33b-5p are usually downregulated in CSCs, and its induced overexpression suppresses the stemness and tumorigenicity by directly binding with the 3′-untranslated region of mitogen-activated protein kinase 8 (MAP3K8) as knockdown of MAP3K8 also suppressed CD90 + CSC characteristics [181]. Activated Wnt/β-catenin signaling in LCSCs is the major causes of therapy resistance in HCC, and it was found that miR-1246 promotes cancer stemness features including resistance by activation of the Wnt/β-catenin pathway [182]. miR-612 negatively regulated stem cell-like properties and tumor metastasis of HCC as miR-612 inversely modulates the mRNA and protein levels of epithelial cell adhesion molecule as well as CD133 negatively regulates the numbers and sizes of tumor spheres, directly inhibits the protein level of Sp1, and subsequently reduces transcription activity of Nanog. Of importance, the higher levels of Sp1 and Nanog in biopsies are the more unfavorable prognoses [183]. Enrichment of stem-like HCC cells by serial passages of hepatospheres with anticancer drugs results into the enhanced stemness features, chemoresistance, self-renewal, tumorigenicity, and metastasis with overexpressed miR-452 which is associated with poor survival of HCC patients and promotes stemness features of HCC [184]. Sorafenib, which is the first-line drug for HCC treatment, often develops resistance and expression of liver-specific miR-122, which is significantly reduced in sorafenib-resistant cells as overexpression of miR-122 overcomes the sorafenib resistance and induction of apoptosis via modulating RAS/RAF/ERK signaling [111].

### 4.8. Head and Neck

Head and neck cancer (HNC), also known as the cancer of the aerodigestive tract, is the sixth most common type of cancer around the world, and more than 90% of reported cases are squamous cell carcinoma (SCCs). Use of tobacco products and alcohol is the prime etiological factors of Head and neck squamous cell carcinoma (HNSCC) [185]. Furthermore, 30–50% of these cases are associated with the infection of human papillomavirus (HPV) [186]. Extensively available findings demonstrate that a number of dysregulated signaling proteins and other factors related to CSCs are the prime causes for the HNSCC pathogenesis and disease recurrence. Various studies have demonstrated the role of aberrant miRNAs such as miR-21, let-7, miR-107, miR-138, and miR-200c, etc. in the development of HNC-CSCs expressing stemness features by modulating signaling pathways [187].

Recently, it has been demonstrated by Li et al. that dysregulated expression of miR-218-5p triggers the metastasis of oral squamous cell carcinoma cells through CD44-ROCK signaling activation [112]. In oral cancer cells, upregulated expression of miR-145 causes reduction in cancer stemness features, including self-renewal and invasion and CD44 expression, suggesting the critical role of miR-145 in oral cancer pathogenesis [188]. Let-7c, an important tumor suppressor miRNA, has been shown to play a vital role in oral cancer resistance and stemness as upregulation of let-7c inhibits the stemness features and sensitizes the cells towards therapy by downregulation of interleukin-8 [189]. Aberrant expression and functioning of miR-21 is critically associated with HNC oncogenesis and development and maintenance of stemness. Mechanistically, the deregulated miR-21 expression is regulated by hyaluronan (HA)/CD44-mediated Nanog/Stat-3 signaling pathways. Further, it has been found that HA/CD44 activates Nanog-Stat-3 signaling, leading to miR-21 expression and silencing, or that knockdown of Nanog/Stat-3 signaling results in decreased miR-21 expression and enhanced chemotherapeutic response [113].

Chang et al. reported that MiR-494 overexpression decreased the ability of sphere formation, invasion, and side population in ALDH1^+^/CD44^+^ head and neck CSCs. Further, they also observed that silibinin treatment-inhibited cell proliferation in ALDH1^+^/CD44^+^ head and neck CSCs reduced sphere formation and stemness features by upregulating miR-494 expression in addition to sensitization towards chemotherapeutic drugs. Overall, their findings concluded the role of miR-494 in reducing cancer stemness of HNC and sensitizing cancer cells to chemotherapy [190]. In another study, Yu et al. demonstrated that knockdown of miR-145 increased the ability of sphere formation and ALDH1^+^/CD44^+^ percentage in ALDH1^−^/CD44^−^ oral cancer cells and elevated the rate of cell proliferation, tumor growth, and invasion, suggesting that miR-145 suppression causes head and neck cancer cells to gain CSCs properties. On the other hand, overexpression of miR-145 in ALDH1^+^/CD44^+^ head and neck cancer cells reduced the stemness features [114]. Moreover, the results revealed that ADAM17 and SOX9 are miR-145 targets and that their expression levels were high in ALDH1^+^/CD44^+^ HNC cells compared to ALDH1^−^/CD44^−^ cells. MiR-145 overexpression in ALDH1^+^/CD44^+^-HNC reduced the levels of ADAM17 and SOX9, while its knocking down increased their expression in ALDH1^−^/CD44^−^ cells. The knocking down of ADAM17 and SOX9 in ALDH1^+^/CD44^+^ HNC cells decreased in the ability of sphere formation and the percentage of ALDH1^+^/CD44^+^ HNC cells and decreased invasion capacity. Collectively, these data reported that miR-145 targets directly ADAM17 and SOX9 to repress their expression and to suppress CSC properties in ALDH1^+^/CD44^+^ HNC cells [114]. Another study by Lo et. al. showed that the critical role of miR-200c in isolated ALDH1^+^/CD44^+^ HNC cells exhibit sphere formation ability. The MiR-200c expression level was found to be significantly reduced in ALDH1^+^/CD44^+^ cells compared to ALDH1^−^/CD44^−^ cells where miR-200c was highly expressed. BMI1 is negatively correlated with miR-200c. BMI1 was found to be highly expressed in ALDH1^+^/CD44^+^, and it is associated with colony formation and invasion. Overexpression of miR-200c in ALDH1^+^/CD44^+^ reduced the BMI expression level as well as stem cell markers including ALDH1, Nanog, Oct4, and SOX2. These results revealed that miR-200c regulates properties of cancer stemness via inhibiting BMI1 expression. Moreover, Overexpression of miR-200c in ALDH1^+^/CD44^+^ sensitized the cells to chemotherapeutic drugs including 5-fluorouracil, cisplatin, doxorubicin, and taxol, which are currently used for HNSCC treatment [115].

### 4.9. Other Human Malignancies

Ovarian cancer (OC) is the most common gynecological malignancy associated with morbidity and mortality among women. OC is an increasing global concern, and despite the extensive studies done in the past years, the clinical consequences have not improved significantly [191]. High morbidity and mortality associated with OC, especially with cases of epithelial OC, have been observed due to diagnostic failures, metastasis, and chemoresistance, and CSCs are found to be the main culprits behind the therapeutic failures and disease heterogeneity and aggressiveness [118,192]. Intense scientific research work and clinical investigations demonstrate that miRNAs are the main molecular regulators of the stemness features related to ovarian CSCs [193].

A recent study shows that deregulated miR-328 overexpression in ovarian CSCs is critical for the maintenance of stemness and tumorigenesis of OC via targeted inhibition of DNA damage-binding protein 2 (DDB2), an inhibitor of stemness in OC. Furthermore, the underlying mechanism suggested that low-level ROSs in ovarian CSCs is the main cause of the downregulation of ERK signaling which led to miR-328 overexpression and degradation of DDB2. Thus, targeting of ROS-ERK-miR-328-DDB2 or mir-328 may lead to eradication of ovarian CSCs and overall clinical improvement in the OC patients [116]. EMT, an important phenomenon associated with stemness-related tumor aggressiveness, has been shown to enhance stemness of OC, and miR-20a and miR-200c can regulate EMT in OC through the regulation of the PI3K/AKT pathway [117]. Another investigation about the underlying mechanisms of miRNA-mediated ovarian tumorigenicity and stemness reveals that miR-628-5p inhibits ovarian CSCs through apoptosis via targeting fibroblast growth factor receptor 2 (FGFR2) [194]. High mortality related to OC is mostly due to stemness-related chemoresistance and metastasis. miRNA-34c-5p has been shown to suppress human amphiregulin (AREG)-induced OC stemness features associated with resistance, relapse, tumorigenesis, and metastasis by downregulation of the AREG-EGFR-ERK pathway, suggesting the crucial role of miRNA-34c-5p and AREG in the OC pathogenesis and that therapeutic complications and targeting may be a viable approach to manage OC [118]. Ovarian cancer spheroids, which contain cells with stemness features known to play a critical role in OC metastasis and drug resistance, has been shown to express constitutively active STAT3-induced transcriptional upregulation of miR-92a, which in turn modulate the Wnt signaling pathway and thus controls the stemness features and metastasis OC [119]. Aberrant dysregulation of Wnt/β-catenin signaling in CSCs is well-known for the development of stemness features, tumorigenesis, and therapeutic resistance in most human cancers, including OC. miR-1207 is often overexpressed in OC, is also associated with poor survival, and has been shown to upregulate the stemness features and tumorigenesis of OC stem cells by activating Wnt/β-catenin signaling via targeted suppression of negative regulators such as SFRP1, AXIN2, β-catenin, and TCF-4 (ICAT) of the Wnt/β-catenin pathway [120]. Another finding shows that miR-17 promotes OC tumorigenesis and pathological manifestations including stemness features and the development of ovarian CSCs from normal ovarian cancer cells by targeting the LKB1-p53-p21/WAF1 signaling pathway [121]. Chemoresistance is a major problem associated with ovarian cancer therapy. The dysregulated suppression of miR-136 in OC SCs has been shown to cause drug resistance via modulating the expression of various proteins associated with stemness and tumorigenesis. Induced overexpression of miR-136 inhibited sphere formation, enhanced the sensitivity of OC cells to chemotherapy, and decreased the expression level of cancer stem cell markers including CD133, CD24, and ALDH1. Further, it reduced the expression level of several genes involved in cell cycle and survival such as *Notch*, *NF-kB*, *Cyclin D1*, *Survivin*, *BCL-XL*, and *BCL2* [122].

Another cancer type which has been a growing concern is the malignancy of the endocrine gland the thyroid. The incidence of thyroid cancer has increased dramatically in recent years. Anaplastic thyroid carcinoma (ATC) accounts for 14–39% of all reported deaths due to thyroid cancer. ATC is the most aggressive type of thyroid cancer and is more metastatic, invasive, and resistant to therapies [124]. The relation between miRNAs and thyroid CSCs is not yet well-known and needs more exploring, and only a few studies have reported the role of miRNAs in thyroid CSC regulation and development. Haghpanah et al. studied the role of knocking down miR-21 on cancer stemness in SW1735 and C643 thyroid cancer cell lines. They found that knocking out miR-21 caused a significant decrease in the expression levels of two CSCs markers, ABCG2 and Oct4. As a result, the differentiation of SW1735 and C643 cell lines is more induced and it is thought to enhance their sensitivity to chemotherapies [123]. Another study showed that suppressed miR-148a expression in ATC-CSCs is critical for the stemness features and tumor formation as the re-expression of miR-148a promoted G2/M cell cycle arrest and inhibited stemness of ATC-CSCs [124].

## 5. Conclusions and Future Prospects

It is now well-established that tumors/cancer of various tissue initiate and harbor from a relatively small population of cells known as CSCs, which are the main oncogenic driving cells due to their self-renewal ability and potential to redevelop entire tumor heterogenicities and, hence, are an important target for the treatment of human malignancies. Enormous findings reveal that CSCs are the main cause for cancer development, metastasis, and drug resistance. A number of dysregulated signaling mechanisms including Notch, Wnt/B-Catenin, JAK/STAT, PI3K/AKT-mTOR, etc., responsible for carcinogenesis and poor clinical outcomes, have been detected in CSCs. Furthermore, identification of protein markers such as ALDH1A1, Sox2, CD44, Oct4, CD133, CD24, and Nanog, etc. have been vital in studying CSCs in cancer research.

Although various mechanisms have been postulated for the maintenance of stemness features and the functioning of CSCs attributed to the oncogenic potential as demonstrated by the studies exploiting the best available experimental conditions to decipher the critical role of CSCs in oncogenesis and poor therapeutic outcomes, the outcomes are not satisfactory and need to be investigated further. The recent development in cancer research has revealed that miRNAs are critical molecules in driving oncogenic potential and therapeutic hindrance due to CSCs and are considered a promising target in cancer therapy. miRNAs are the small noncoding nucleic acids that negatively regulate gene expression post-transcriptionally. Deregulated expression and functioning of miRNAs have been considered an important mechanism in various pathological conditions including CSCs in cancer as a single miRNA can control the expression of multiple genes. In this review, we discussed the role of miRNAs in regulating CSCs and associated challenges in the understanding of cancer development and therapeutics. Now, it is well-established that aberrant or dysregulated functioning of miRNAs is the leading cause for the growth and maintenance of CSCs in various malignancies and drawbacks related to conventional therapeutic measures. Hence, targeting of miRNA can be vital for the elimination of CSCs and associated challenges like cancer recurrence, resistance, and metastasis as miRNAs are known to have regulatory action on different properties of CSCs. Many interesting and important discoveries have been achieved on the role of miRNA in many diseases including cancer cells during the last decade. Early detection, effective therapeutics, and cures are goals that drive research by many investigators. miRNAs, with known sequences, are well conserved between species, making it an attractive therapeutic target for the treatment and management of cancers. Therapeutic potentials could be exploited in cancer cells expressing an aberrant level of miRNA. Many studies are now providing evidence that miRNAs are involved in the regulation of cancer stem cells and its regulated self-renewal process. In addition to the functional role of miRNA, the therapeutic potentials of miRNAs are getting attention by many researchers and clinicians. A number of mimics and antagonists of miRNAs are currently being investigated for clinical application. Interestingly, miRNAs have been proposed as targets as well as therapeutics to contribute to enhancing the combined treatment of cancer with other modality. A number of translational studies have shown that the combined potential of miRNA and anticancer agents showed a positive outcome at the preclinical level. It has been shown that the expression of miR-210 was downregulated in relapsed acute lymphoblastic leukemia (ALL) patients as compared to other patients [195]. Interestingly, overexpression of miR-210 augmented the response of ALL cells to daunorubicin-dexamethasone-L-asparaginase and daunorubicin-dexamethasone-L-vincristine combinations. A similar observation was reported in which the forced overexpression of miR-210 increased the temozolomide sensitivity in glioblastoma cells [196]. These findings strongly implicate the role of miR-210 as a biomarker for the prediction of drug sensitivity. Furthermore, it also indicates the therapeutic potential of miR-210 in chemosensitization. Many pharma companies are actively involved in a clinical trial in miRNA-based therapy. Although the discovery of miRNA has enormous potential as a biomarker and therapeutic potential for the treatment of many diseases, it has many open questions to reach its application. miRNA-based drugs are still in its clinical trials phase, and none have reached its final application. The delivery miRNA system to target cells is yet to be standardized. The immunogenic and toxic effects of miRNA have to be determined in target cells. The optimization of doses and combination with other conventional drugs has to be optimized using various in vivo and clinical models. Future development and deep mechanistic understandings may shed new insights about the involvement of miRNAs and CSCs in human carcinogenesis and challenges of cancer therapy that may lead to a better way for the targeted eradication of CSCs and its regulators and, hence, can be a future potential tool for cancer management and therapy. Altogether, remarkable development and efforts have been made to bring the miRNA-based therapy from the lab to the clinic.

## Figures and Tables

**Table 1:** The regulatory roles of miRNA in CSCs.

Cancer Type	miRNA	Signaling Pathways/Targeting Gene	References
Colorectal	miR-21	PTEN, AKT, Ras	[58]
	MiRNA-215	DTL	[59]
	miR-148a	WNT, β-catenin	[60]
	miR-199a/b	Gsk3β, Wnt/β-catenin-ABCG2	[61]
	miR-196b-5p	STAT3	[62]
	miRs-31	EphB2 and EphA2	[63]
	miR-27a	Apaf-1/caspase-9	[64]
	miR-372/373	Nanog, Hedgehog, NFκB, MAPK/Erk, VDR Jak-STAT, TGF-beta, PI3K-Akt, MAPK.	[65,66]
	miR-137	DCLK1	[67]
	miR-146a	β-catenin	[68]
	miR-195-5p	STAT3, BIRC5, BCL2, BCL-XL, SOX2, CD133, RBPJ, Notch2	[62,69]
Lung	miR-128	AKT/ERK, p38, c-met/PI3K/AKT, VEGF/PI3K/AKT, IL-6-JAK-STAT3	[70,71,72,73]
	miR-181b	Notch2	[74]
	miR-138	TGFβ	[75]
	miR-5100	Rab6	[76]
	miR-214	c-MYC	[76]
	miR-708-5p	Wnt/β-catenin	[77]
	miR-873 miR-125a-3p	Oct4, Nanog and ALDH	[78]
	miR-23a	PTEN/PI3K/Akt	[79]
	miR-494-3p	NOTCH1-PI3K	[80]
	miR-19a/19b	Wnt/β-catenin	[81]
Breast	miR-1287-5p	PI3Kinase	[82]
	miR-137	β3/Wnt, BCL11A	[83,84]
	miR-34a	Wnt/beta-catenin	[85]
	miRNA-140-5p	Wnt/β-catenin	[86]
	miR205	STAT3	[87]
	miR-31	Prlr/Stat5, TGFβ and Wnt/β-catenin	[88]
	miR-221/222	ALDH1, PTEN, p65, pp65, p-AKT, COX-2	[89]
	miR-519d	Bcl-2, MCL-1	[90]
Gastric	MiR-26a	HOXC9	[91]
	miRNA-19b/20a/92a	β-catenin	[92]
	miR-483-5p	Wnt/β-catenin	[93]
	miR-106b	TGF-β/Smad	[94]
	miR-132	SIRT1/CREB/ABCG2	[95]
	miR-501-5p	DKK1, NKD1, GSK3β, IL-6R/STAT3	[96,97]
Prostate	miR-218	OCT4, SOX2, NANOG, CD44, KLF4, c-MYC, Wnt,	[98]
	miR-424	STAT3	[99]
	miR-302/367	NANOG, SOX2, OCT4, KLF4, BMI-1, LATS2/YAP	[100]
	miR-199a-3p	c-MYC, cyclin D1 (CCND1), EGFR	[101]
	miR-143	MMP-9, MMP-2	[102]
	miR-7	KLF4/PI3K/Akt/p21	[103]
Pancreatic	miR-100 miR-125b	TGF-β	[104]
	miR-200c	Notch1	[105]
Liver	miR-6875-3p	BTG2/FAK/Akt	[106]
	miR-106b-5p	PI3K/Akt	[107]
	miR-191	HIF-2α	[108]
	miR-217	Wnt	[109]
	miR-500a-3p	SOCS2, SOCS4, PTPN11, STAT3	[110]
	miR-122	RAS/RAF/ERK	[111]
Head and Neck	miR-218-5p	CD44-ROCK	[112]
	Mir-21	Nanog-Stat-3	[113]
	miR-145	ADAM17, SOX9	[114]
	miR-200c	ALDH1, Nanog, Oct4, SOX2.	[115]
Ovarian	miR-328	ERK	[116]
	miR-20a miR-200c	PI3K/AKT	[117]
	miRNA-34c-5p	EGFR-ERK	[118]
	miR-92a	Wnt	[119]
	miR-1207	Wnt/β-catenin	[120]
	miR-17	LKB1-p53-p21/WAF1	[121]
	miR-136	NOTCH3, NF-kB, Cyclin D1, Survivin, BCL-XL, BCL2	[122]
Thyroid	miR-21	ABCG2, Oct4	[123]
	miR-148a	ATC-CSCs	[124]

## Abbreviations

microRNAs	miRNAs
Cancer stem cells	CSCs
Prostate cancer	PCa
colorectal cancer	CRC
untranslated region	UTR
5-fluorouracil	FU
tumor microenvironment	TME
epithelial to mesenchymal transition	EMT
transforming growth factor β	TGFβ
head and neck cancer	HNSC
aldehyde dehydrogenase1	ALDH1
Janus-activated kinase/signal transducer and activator of transcription	JAK/STAT
phosphatidylinositol 3-kinase/phosphatase and tensin homolog	PI3K/PTEN
nuclear factor-kB	NF- kB
triple negative breast cancer	TNBC
gastric cancer	GC
